# Managing Adhesive Small Bowel Obstruction with Water-Soluble Contrast Should Be Protocolized: A Retrospective Analysis

**DOI:** 10.1055/s-0038-1666781

**Published:** 2018-07-20

**Authors:** Jakob K. Köstenbauer

**Affiliations:** 1 Department of Surgery, University of New South Wales, Wagga Wagga Rural Referral Hospital, Wagga Wagga, New South Wales, Australia

**Keywords:** Gastrografin, water-soluble contrast agents, adhesions, small bowel obstruction, management protocol, computed tomography

## Abstract

**Aim** Current literature emphasizes the effectiveness of computed tomography (CT) and water-soluble contrast agent, Gastrografin, in the investigation of adhesive small bowel obstruction (ASBO). As there is no management protocol for ASBO at our institution, the aim of this study was to determine the effect of imaging methods—CT, Gastrografin challenge (GC), or plain-film X-ray—on patient outcomes in a clinical setting.

**Methods** All 163 emergency presentations of ASBO during the study period between December 2010 and September 2012 were collected retrospectively. Cases were divided into three groups: CT with oral contrast, GC, or plain-film X-ray only. The primary outcome was time to theater.

**Results** Patients investigated with X-ray only were significantly less likely to require surgery (6% in plain-film X-ray vs. 35% and 20% in CT and GC, respectively; *p* = 0.003). In cases requiring surgery, GC was associated with a 24-hour longer time to imaging than CT ( *p* < 0.001). The time to theater was 71:25 hours for GC versus 46:39 for CT ( *p* = 0.039). There was no significant difference in bowel resection or complication rates.

**Conclusion** Patients undergoing water-soluble contrast studies were subjected to unnecessary delays in their clinical course. These delays are costly and avoidable. The development and implementation of an evidence-based protocol for the management of small bowel obstruction is strongly recommended. The lack of a protocol likely caused significant delays in Gastrografin administration, reducing its known benefits for clinical decision-making and length of stay.

Small bowel obstruction (SBO) is a common yet complex problem for general surgeons the world over. Adhesive SBO (ASBO) accounts for up to 15% of all surgical presentations and has an estimated annual incidence of 20 cases per 100,000 adults in the United States. 1 2 The optimal assessment, management, and treatment of ASBO is still evolving and greatly debated. 3 4

There are three imaging modalities used for the diagnosis of ASBO: plain-film (PF) X-ray, computed tomography (CT), or a Gastrografin challenge (GC). Gastrografin is a hyperosmolar solution that, when given orally and undiluted in volumes of 100 mL, can accurately diagnose SBO, predict the need for surgery, reduce the need for surgery, and reduce hospital stay in uncomplicated SBO. 5 6 This is referred to as a “Gastrografin challenge.” Gastrografin in a diluted concentration, but high volume (4%/500 mL) is also commonly used in CT enterography in the diagnosis of acute SBO. A challenge faced by many general surgeons is the integration of CT and/or GCs into the management plan. In theory, an ideal management strategy would enable the timely prediction and acceleration of successful conservative treatment, while reducing the operative delay for cases of strangulated bowel.

There are currently no set protocols regarding the use or dosing of Gastrografin at our institution. The aim of this study was therefore to determine the relationship between the imaging modality used for work-up and patient outcomes. Given the recent evidence suggesting the ability of a GC to reduce the need for surgery and overall length of stay, 5 6 the author hypothesized that patients receiving higher-dose Gastrografin would have a decreased need for operation, time to operation, and length of stay.

## Methods

A retrospective analysis of emergency presentations to our hospital with ASBO was conducted. Ethical approval was granted by the Human Research Ethics Committee of the Area Health Service.

### Cohort

A search of the hospital administrative database for all cases of SBO between December 2010 and September 2012 identified 454 cases. Of these, 184 were confirmed as emergency presentations with ASBO. Cases did not match the inclusion criteria due to coding error, nonadhesive etiology, or SBO during hospital admission. Of the 184 confirmed cases of ASBO, 8 were excluded for undergoing abdominal CT without the use of oral contrast, 2 for undergoing incomplete GC when Gastrografin was not tolerated, 5 due to incomplete or erroneous records, 3 for admission as elective surgery, and 3 for early discharge against medical advice. A total of 163 cases were included in this study.

### Patient Characteristics

Patient characteristics and management data were collected from patients' medical records ( Table 1 ). Management data included the time of surgery (by start time of operation), time and type of imaging, and date of discharge. The time of day at admission, imaging, and operation were defined as day-time hours (08:00–20:00 hours) or after-hours (20:00–08:00 hours).

**Table 1:** Cohort demographics and management path

	Total cohort ( *n* = 163)
Median age in years (range)	74 (26–97)
Female gender	95 (58%)
After-hours admission	66 (40%)
High-dose group	45 (28%)
Low-dose group	85 (52%)
Plain-film X-ray	33 (20%)
Required surgery	41 (25%)

### Independent Exposure Variable

Patients were grouped into one of three groups based on oral contrast dose: GC, CT with oral contrast, or PF only. The GC group included all patients whose records indicated they had received and tolerated ≥100 mL of oral Gastrografin for a GC. This group also included patients who received dilute oral contrast for a CT scan prior to high-dose GCs. The low-dose CT group consisted of patients who received dilute oral Gastrografin (20-mL Gastrografin in 500 mL of water) prior to CT scanning. Patients who underwent neither CT nor GC were assessed with PF radiographs only, received no oral contrast, and were defined as PF.

### Primary Outcome

The primary outcome was time to theater, defined as time from admission to the commencement of operation.

### Secondary Outcomes

Secondary outcomes for this study were need for surgery, overall length of stay, inhospital postoperative complications, mortality, time from admission to imaging, postoperative length of stay (PLOS) and readmission with ASBO. Need for operation was defined as whether or not patients received surgery for ASBO during their admission. Overall length of stay (days) was calculated from the date of admission to the date of discharge. Classification of postoperative complications was determined a priori and included all causes. Mortality was defined as death during admission or within 30 days of discharge. Time from admission to imaging was defined as time from triage to the start of imaging. The PLOS was defined as the difference between the recorded date of surgery and the recorded date of discharge. Readmission was divided into those readmitted with ASBO within 30 days and those within 12 months.

### Statistical Methods

Categorical variables were analyzed using the chi-square test for independence. Yates' Continuity Correction values were used to compensate for any potential overestimation of the chi-square value when used with 2 × 2 tables. Where 2 × 2 tables had an expected frequency less than 10, Fisher's exact test was used. All significance levels of Fisher's exact tests were two-tailed. Where 2 × 3 tables were used, Pearson's chi-square test was used. Continuous variables were analyzed using the Mann–Whitney *U* test, when comparing two variables, or the Kruskal–Wallis test, when comparing three or more variables, due to the abnormal distribution of the data. The cutoff for statistical significance was defined as *p* < 0.05. All data were recorded in Microsoft Excel and analyzed using SPSS version 21 (SPSS Inc., Chicago, IL).

## Results

An overview of the cohort's demographics and management paths are illustrated in Tables 1 and 2 . The GC group consisted of 45 cases, of which 32 had undergone prior CT scanning. Patient comorbidities and surgical histories were compared across the three imaging groups ( Table 3 ). Analysis showed no statistically significant differences in age, gender, and after-hours admission. Patients managed using only PF radiography were significantly less likely to require surgery (6% in PF vs. 35% and 20% in CT and GC, respectively) and had significantly shorter lengths of stay (2 days in PF versus 5 days (CT) or 4 days (GC); *p* < 0.001).

**Table 2:** Patient factors associated with surgery

	No surgery ( *n* = 122)	Surgery ( *n* = 41)	*p* -Value
Female gender	68 (56%)	27 (66%)	0.34
After-hours admission	46 (38%)	19 (46%)	0.45
Previous history of SBO	61 (50%)	10 (24%)	0.007
Previous abdominal or pelvic surgery	121 (99%)	40 (98%)	0.44 a
Diabetes mellitus	18 (15%)	7 (17%)	0.80
Cardiovascular comorbidities	71 (58%)	30 (73%)	0.13
Previous abdominal irradiation	20 (16%)	2 (5%)	0.11
Previous abdominal/pelvic malignancy	54 (44%)	11 (27%)	0.07
Anticoagulation	8 (7%)	6 (15%)	0.12 a

Abbreviation: SBO, small bowel obstruction.

a Fisher's exact test.

**Table 3:** Patient demographics and management by imaging modality

	CT with oral contrast ( *n* = 85)	Gastrografin challenge ( *n* = 45)	Neither ( *n* = 33)	*p* -Value a
Median age in years (range)	76 (26–97)	67 (28–90)	64 (27–91)	0.08
Female gender	46 (54%)	27 (60%)	22 (67%)	0.45
After-hours admission	31 (36%)	16 (36%)	19 (58%)	0.08
Previous abdominal or pelvic operation	83 (97%)	45 (100%)	33 (100%)	0.40
History of previous SBO	26 (31%)	19 (42%)	26 (78%)	< 0.001
Cardiovascular comorbidities	60 (71%)	27 (60%)	14 (42%)	0.017
Diabetes mellitus	18 (21%)	6 (13%)	1 (3%)	0.045
Previous abdominal irradiation	10 (12%)	5 (11%)	7 (21%)	0.35
Previous abdominal/pelvic malignancy	30 (35%)	21 (47%)	14 (42%)	0.43
Anticoagulation	5 (6%)	6 (13%)	3 (9%)	0.35
Surgery required	30 (35%)	9 (20%)	2 (6%)	0.003
Median length of stay in days (range)	5 (1–68)	4 (1–42)	2 (1–19)	< 0.001

Abbreviations: CT, computed tomography; SBO, small bowel obstruction.

a Pearson's chi-square test used for categorical variables and the Kruskal–Wallis test used for continuous variables.

Patients undergoing GC had a significantly increased time from admission to imaging (Mann–Whitney *U* test; Z = –3.83; *p* < 0.001). This delay was accompanied by a corresponding longer time to theater for the GC group (71:25 hours in GC vs. 46:39 hours in CT). There was no significant difference in complication rates.

Of the 41 admissions requiring surgery, only 2 were evaluated with PF. This number did not allow for accurate analysis without introducing significant error. Further analyses of imaging modality in the operative cases therefore include only CT and GC groups, with a total of 39 surgical cases ( Table 4 ). There was no significant difference in demographic factors between the CT and GC patients who required surgery. All patients in the GC group were operated on during day-time hours compared with 20 (67%) in the CT group. This difference was not statistically significant ( *p* = 0.079). Of the 117 cases investigated with CT scanning, 33 (28%) were performed after-hours, whereas all GCs were performed during day-time hours.

**Table 4:** Demographics and outcomes of patients who underwent surgery

	CT with oral contrast ( *n* = 30)	Gastrografin challenge ( *n* = 9)	*p* -Value
Median age in years (range)	79 (43–90)	79 (38–86)	0.53
Female gender	18 (60%)	7 (78%)	0.56
Operation during day-time	20 (67%)	9 (100%)	0.08 a
Median length of stay in days (range)	10 (2–68)	14 (7–42)	0.26
Median time to imaging in hours:minutes	7:55	32:24	<0.001
Median days from imaging to discharge (range)	10 (2–68)	13 (6–41)	0.61
Median time to theater in hours:minutes	46:39	71:25	0.039
Surgery > 48 h after admission	14 (47%)	8 (89%)	0.052 a
Median postoperative days (range)	8 (1–65)	9 (4–39)	0.59
Bowel resection	11 (37%)	4 (44%)	0.71 a
Number of patients with complications	13 (43%)	7 (78%)	0.13 a
Readmission within 30 d	0	1 (11%)	0.23 a
Readmission within 12 mo	5 (17%)	1 (11%)	1.00 a

Abbreviation: CT, computed tomography.

a Fisher's exact test.

All patients had a history of previous abdominal or pelvic surgery, with the exception of two patients in the CT group, who had known adhesions from abdominal trauma after motor-vehicle accidents. Patients in the PF group were more likely to have a recorded previous history of ASBO ( *p* < 0.001). They were also significantly less likely to have cardiovascular comorbidities or diabetes mellitus.

Readmission rates were not associated with different oral contrast doses, regardless of whether patients underwent operative or conservative management. Surgically managed patients were, however, 11.6% less likely to require readmission within 30 days than conservatively managed patients ( Table 4 ). There was no difference in readmission within 12 months (17% of operative vs. 20% of conservative; *p* = 0.891).

For those managed conservatively, the median time from admission to imaging was increased by more than 20 hours in the GC group. Imaging modality had no effect on the postimaging length of stay nor the overall length of stay of conservatively treated patients ( Table 5 ). No data were available regarding the timing of PF imaging.

**Table 5:** Demographics and outcomes of patients who underwent conservative management

	CT with oral contrast ( *n* = 55)	Gastrografin challenge ( *n* = 36)	Neither ( *n* = 31)	*p* -Value
Median age in years (range)	74 (26–97)	67 (28–90)	64 (27–91)	0.27
Female gender	28 (51%)	20 (56%)	20 (65%)	0.83
Median time to imaging in hours:minutes	5:27	25:50	n/a	<0.001
Median days from imaging to discharge (range)	3 (0–19)	3 (1–126)	n/a	0.86
Median length of stay in days (range)	3 (1–20)	4 (1–10)	2 (1–6)	<0.001
Readmission within 30 d	7 (13%)	5 (14%)	5 (16%)	0.91
Readmission within 12 mo	8 (15%)	10 (28%)	6 (19%)	0.20

Abbreviation: CT, computed tomography.

The most common surgical complication was bowel resection, occurring in 15 (38%) of the 39 surgical cases. Cardiac and respiratory complications were the most common general postoperative complications ( Table 6 ). In this study, there was a single mortality (in the CT group). The mortality was a conservatively managed, 83-year-old male with preexisting cardiovascular disease and dementia who succumbed to pneumonia.

**Table 6:** Complications of patients who underwent surgery

	CT with oral contrast ( *n* = 30)	Gastrografin challenge ( *n* = 9)	*p* -Value a
Number of patients with complications ^b^	13	7	0.13
Bowel resection	11	4	0.71
Wound infection	1	0	1.00
Wound dehiscence	2	0	0.49
Fistula	0	0	0
Fluid collection	1	0	1.00
Respiratory	6	1	0.13
Urinary	2	1	1.00
Line complications	1	0	1.00
Other infectious	4	1	1.00
Pseudocolitis	0	0	0
Cardiac	5	2	0.62
Electrolyte and metabolic complication	3	2	1.00

a Fisher's Exact Test.

## Discussion

This study was a retrospective review of the use of three imaging modalities used to investigate ASBO. The current implementation of GC at our institution did not accelerate surgical decision-making. In direct contrast to the hypothesis, the GC group had significantly longer time to theater (71 vs. 47 hours) and time to imaging.

The majority of patients with ASBO do not display overt signs of strangulation and often pose the greatest challenge to the surgeon as these patients may harbor silent strangulation or develop ischaemia during conservative management. In this group, delay of treatment is known to be detrimental. 7 8 9 10 However, to avoid nontherapeutic laparotomies in the majority of patients who do not have strangulation, a trial of conservative management between 3 and to 5 days is widely accepted as appropriate. 3 4 11

Water-soluble contrast studies have proven to be highly accurate in identifying patients likely to fail a trial of conservative management. 5 6 Recently, CT scanning has been recommended as the primary imaging modality in all patients presenting with suspected SBO. A CT scan is the most accurate method in confirming diagnosis and etiology of SBO and is useful in rapidly identifying patients unsuitable for a trial of conservative management. 4 12 13 14 This is in contrast to previous reviews and randomized trials, which have advocated the use of CT as an adjunct to PF radiography and to be used only in cases where PF and clinical findings are ambiguous. 3 15 16 17

Of the total 163 patient cohort, the proportion of patients undergoing a GC (28%) was far less than the proportion of those managed conservatively (76%). These proportions were expected to be similar as GC is highly accurate in confirming the suitability of conservative management and accelerates resolution of symptoms. 6 There are several explanations for this difference, as well as for the delay in time to imaging and theater in the high-dose group. First, surgeons may be hesitant to operate on high-risk patients, thus delaying surgery to allow more time for an obstruction to resolve nonoperatively. This is supported by the results, which demonstrated a corresponding difference in comorbidities between the PF group and the GC group. Second, increased delay in a patient's presentation to hospital with ASBO is a recognized risk factor for increased PLOS and morbidity. 10 Unfortunately, this study did not include data on the time to presentation. At our institution Gastrografin was not available after-hours during the trial period, whereas CT is available at all times (in this study 28% were performed after-hours). For this reason, patients deemed suitable for conservative management may have undergone delayed GC or may have been preferentially investigated with CT or PF only.

Patients in the PF group had less comorbidities, and 78% had a previous history of ASBO. It is possible therefore that this group constitutes patients familiar with the early symptoms of ASBO or those who had clinically obvious, incomplete obstruction. Conservative management of cases in which the diagnosis is confirmed as incomplete or resolving SBO can be safely managed without the use of GC. As a result, the patients in the PF group were treated and discharged significantly faster without increased rates of silent strangulation or early readmission. It must, however, be said that this approach cannot match the predictive accuracy of GC (99%) reported in the literature. 5 6 It is possible that the two patients of the PF group who required surgery could have undergone earlier decision-making or avoided surgery if GC were implemented for all conservative patients. Their median time to theater was 94 hours, and they had a total length of stay of 18 and 19 days, respectively.

Female gender (66%; *p* = 0.34) and no history of SBO (76%; *p* = 0.007) were positively associated with surgery ( Table 2 ). This observation is consistent with the literature 18 and may be explained by the increased rate of pelvic surgery amongst females as ASBO involving pelvic adhesions is more likely to require surgical intervention. 19

The overall operative rate of 24% (39 of 163) was comparable to recent literature, in which rates ranged from 17 to 24%. 18 20 21 The resection rate of 37% is also comparable to the reported range of 25 to 45%. 9 18 The observed median time to theater of 47 hours (CT) and 71 hours (GC) was similar to the range—48 hours (for CT) and 72 hours (for GC)—reported by Goussous et al. 12 The overall median time to theater reported in the literature range from 24 hours to 5 days. 7 9 20 22 The median total length of stay of 12 days for surgically managed and 3 days for conservative patients was in the high end of the range reported in the literature (8.5–12 days vs. 3–5 days, respectively). 18 22 23

Operative management, irrespective of imaging pathway, resulted in 11.6% fewer readmissions within 30 days, although no difference was demonstrated in late readmissions (within 12 months). Lower readmission rates among surgically treated patients have been widely reported in the literature, 11 with conservative management resulting in a higher readmission rate (40 vs. 20–27%) and shorter time to readmission (153 vs. 411 days). 22 23 24 This difference is likely due to a relatively short 12-month cutoff, rather than 5- to 10-year cutoffs used in previous studies. That said, these findings certainly do not warrant performing operations on more patients in an attempt to reduce recurrence, as cumulative inhospital stay, morbidity, and mortality remain lower in conservatively managed patients. 11 22 23

This study identified substantial methodological variations in the execution of GC. The timing of follow-up radiographs varied by 20 hours, whereas the dosing of contrast and the total number of radiographs looking for contrast in the colon also varied significantly. These differences in methodology are likely due to the lack of an institution-wide protocol detailing the optimal use of GC for the assessment of SBO.

## Recommendations

The inconsistencies described in this study contribute to the suboptimal results of using GC as a management tool, particularly the delays associated with its use. An externally validated, evidence-based, and cost-effective management protocol for SBO should be implemented in all major hospitals to reduce the time to theater, overall length of stay, costs, and failure rates of conservative management. A protocol should enable the integrated and combined use of a predictive CT algorithm and GC test. The feasibility of this approach has been highlighted in recent best-practice guidelines and several key studies. 3 4 11 25 26 27 In this study, there was also no increase in complications between imaging groups, despite increased time to theater for GC, although PLOS, and likely costs, were increased. Implementation of any integrative protocol can be safe and can effectively reduce delays.

## Limitations

This study's limitations were primarily those inherent to a retrospective study design. The study relied on sourcing data from the institution's administrative database, which relies on disease codes determined at a patient's initial presentation. The accuracy of data was therefore limited to that of the available medical records. Detailed clinical findings such as duration of symptoms and practitioner preferences were not always available, which may have contributed as significant confounding factors. The cohort's size was not large enough to allow for statistically significant comparisons between certain groups, for example, the outcomes of surgical patients in the PF group ( *n* = 2), nor did the numbers enable more powerful statistical tests such as regression analyses or propensity score methods. Viewed within the limitations of this retrospective study, the findings likely identify a pattern of delays common to many similar metropolitan institutions, though the exact extents are unlikely to be directly replicable.

## Conclusion

The use of a GC in cases of ASBO is associated with significant delays in time to imaging and time to theater at our institution. Despite the safety of current practice, treatment delays result in increased length of stay and costs. There is a great need for a protocol which incorporates the latest evidence on water-soluble contrast agents and CT-based algorithms into an inclusive evidence-based guideline to reduce delays and improve outcomes.

## References

[1] Ray N F Denton W G Thamer M Henderson S C Perry S Abdominal adhesiolysis: inpatient care and expenditures in the United States in 1994 J Am Coll Surg 1998 186 01 1 9 9449594 10.1016/s1072-7515(97)00127-0

[2] Geiger T M Roberts P L Read T E Marcello P W Schoetz D J Ricciardi R Has the use of anti-adhesion barriers affected the national rate of bowel obstruction? Am Surg 2011 77 06 773 777 21679649

[3] Diaz J JJ Jr Bokhari F Mowery N T Guidelines for management of small bowel obstruction J Trauma 2008 64 06 1651 1664 18545135 10.1097/TA.0b013e31816f709e

[4] Maung A A Johnson D C Piper G L Evaluation and management of small-bowel obstruction: an Eastern Association for the Surgery of Trauma practice management guideline J Trauma Acute Care Surg 2012 73 05 04 S362 S369 23114494 10.1097/TA.0b013e31827019de

[5] Branco B C Barmparas G Schnüriger B Inaba K Chan L S Demetriades D Systematic review and meta-analysis of the diagnostic and therapeutic role of water-soluble contrast agent in adhesive small bowel obstruction Br J Surg 2010 97 04 470 478 20205228 10.1002/bjs.7019

[6] Ceresoli M Coccolini F Catena F Water-soluble contrast agent in adhesive small bowel obstruction: a systematic review and meta-analysis of diagnostic and therapeutic value Am J Surg 2016 211 06 1114 1125 26329902 10.1016/j.amjsurg.2015.06.012

[7] Fevang B T Jensen D Svanes K Viste A Early operation or conservative management of patients with small bowel obstruction? Eur J Surg 2002 168(8-9):475 481 12549688 10.1080/110241502321116488

[8] Zielinski M D Eiken P W Heller S F Prospective, observational validation of a multivariate small-bowel obstruction model to predict the need for operative intervention J Am Coll Surg 2011 212 06 1068 1076 21458305 10.1016/j.jamcollsurg.2011.02.023

[9] Bickell N A Federman A D Aufses A H Jr Influence of time on risk of bowel resection in complete small bowel obstruction J Am Coll Surg 2005 201 06 847 854 16310687 10.1016/j.jamcollsurg.2005.07.005

[10] Fevang B T Fevang J M Søreide O Svanes K Viste A Delay in operative treatment among patients with small bowel obstruction Scand J Surg 2003 92 02 131 137 12841553 10.1177/145749690309200204

[11] Catena F Di Saverio S Kelly M D Bologna guidelines for diagnosis and management of adhesive small bowel obstruction (ASBO): 2010 evidence-based guidelines of the world society of emergency surgery World J Emerg Surg 2011 6 05 5 21255429 10.1186/1749-7922-6-5 PMC3037327

[12] Goussous N Eiken P W Bannon M P Zielinski M D Enhancement of a small bowel obstruction model using the gastrografin® challenge test J Gastrointest Surg 2013 17 01 110 116, discussion 116–117 22923211 10.1007/s11605-012-2011-6

[13] Zielinski M D Bannon M P Current management of small bowel obstruction Adv Surg 2011 45 01 1 29 21954676 10.1016/j.yasu.2011.03.017

[14] Santillan C S Computed tomography of small bowel obstruction Radiol Clin North Am 2013 51 01 17 27 23182505 10.1016/j.rcl.2012.09.002

[15] Maglinte D D Kelvin F M Sandrasegaran K Radiology of small bowel obstruction: contemporary approach and controversies Abdom Imaging 2005 30 02 160 178 15688118 10.1007/s00261-004-0211-6

[16] Kendrick M L Partial small bowel obstruction: clinical issues and recent technical advances Abdom Imaging 2009 34 03 329 334 18597140 10.1007/s00261-008-9436-0

[17] Trésallet C Lebreton N Royer B Leyre P Godiris-Petit G Menegaux F Improving the management of acute adhesive small bowel obstruction with CT-scan and water-soluble contrast medium: a prospective study Dis Colon Rectum 2009 52 11 1869 1876 19966635 10.1007/DCR.0b013e3181b35c06

[18] Schraufnagel D Rajaee S Millham F H How many sunsets? Timing of surgery in adhesive small bowel obstruction: a study of the Nationwide Inpatient Sample J Trauma Acute Care Surg 2013 74 01 181 187, discussion 187–189 23271094 10.1097/TA.0b013e31827891a1

[19] Ellis H The clinical significance of adhesions: focus on intestinal obstruction Eur J Surg Suppl 1997 577 577 5 9 9076446

[20] Chu D I Gainsbury M L Howard L A Stucchi A F Becker J M Early versus late adhesiolysis for adhesive-related intestinal obstruction: a nationwide analysis of inpatient outcomes J Gastrointest Surg 2013 17 02 288 297 22914981 10.1007/s11605-012-1953-z

[21] Foster N M McGory M L Zingmond D S Ko C Y Small bowel obstruction: a population-based appraisal J Am Coll Surg 2006 203 02 170 176 16864029 10.1016/j.jamcollsurg.2006.04.020

[22] Miller G Boman J Shrier I Gordon P H Natural history of patients with adhesive small bowel obstruction Br J Surg 2000 87 09 1240 1247 10971435 10.1046/j.1365-2168.2000.01530.x

[23] Williams S B Greenspon J Young H A Orkin B A Small bowel obstruction: conservative vs. surgical management Dis Colon Rectum 2005 48 06 1140 1146 15906139 10.1007/s10350-004-0882-7

[24] Barkan H Webster S Ozeran S Factors predicting the recurrence of adhesive small-bowel obstruction Am J Surg 1995 170 04 361 365 7573729 10.1016/s0002-9610(99)80304-3

[25] Jones K Mangram A J Lebron R A Nadalo L Dunn E Can a computed tomography scoring system predict the need for surgery in small-bowel obstruction? Am J Surg 2007 194 06 780 783, discussion 783–784 18005771 10.1016/j.amjsurg.2007.09.020

[26] Schwenter F Poletti P A Platon A Perneger T Morel P Gervaz P Clinicoradiological score for predicting the risk of strangulated small bowel obstruction Br J Surg 2010 97 07 1119 1125 20632281 10.1002/bjs.7037

[27] Zielinski M D Eiken P W Bannon M P Small bowel obstruction-who needs an operation? A multivariate prediction model World J Surg 2010 34 05 910 919 20217412 10.1007/s00268-010-0479-3 PMC4882094

